# The Institutional Library

**Published:** 1916-04-01

**Authors:** 


### THE INSTITUTIONAL LIBRARY.

### THE PRACTICE OF HOSPITAL ADMINISTRATION.

**Construction, Equipment, and Management of a General Hospital.** By Donald Mackintosh, M.V.O., M.B., LL.D., F.R.S.E., Medical Superintendent, Western Infirmary, Glasgow. Second Edition, with Plans and Illustrations. (Edinburgh and London: William Hodge. Price 10s. 6d. net.)

We are glad to welcome a second edition of Dr. Mackintosh's book on "Construction, Equipment, and Management of a General Hospital." This book was reviewed at length in The Hospital, November 27, 1909. The new volume is practically a replica of the first edition, with the addition of a chapter on the construction of hospitals of a semi-permanent nature by Mr. John Wilson, F.R.I.B.A. The title gives an adequate idea of the scope of the volume, the basis being mainly the exposition of the Western Infirmary, Glasgow, its planning and lessons, by an experienced administrator, Dr. Mackintosh, who has been for many years its medical superintendent.

In the preface the author, in a telling phrase, writes "that the theory of hospital construction is a living thing, and it is not possible at any stage to say that a climax of excellence in design has been reached." This was written about seven years ago, but the present edition gives no indication of what has taken place during that period. The book cannot be said to be a manual of hospital planning, but it has one great merit which is not to be found in any work on the subject that we know of, in that it affords much excellent material and guidance in hospital design, based upon the actual experience of medical superintendence, and that is everything.

It is practically impossible for an architect, without matured knowledge of hospital administration, to undertake with safety the design of even a small hospital without long study and reflection over existing hospitals, both good and indifferent in their arrangement. We say this advisedly, for with long experience of hospital visitation throughout the country we are convinced that it is only by the studied inspection of hospitals of all kinds, good and indifferent, combined with the experience of those who work them who will freely give to those who seek suggestive enlightenment, that the knowledge of what should not be found to-day in hospital planning can be learnt and better arrangements suggested.

We commend to the reader's consideration Dr. Mackintosh's telling dictum of "the living thing" of hospital planning; there is still much to do in original development which makes the study and practice of hospital architecture so fascinating. We may next note an additional chapter by Mr. J. B. Wilson, F.R.I.B.A., on the construction of hospitals of a semi-permanent nature, the writer's intention being to describe "the construction of a hospital to remain structurally good for a limited time." What that time should be is not clear, but probably it is for the period of a war such as the present, but the proposals made tend to permanent rather than to temporary hospitals. Eight methods of building construction are set forth in a variety of methods. The writer of the chapter drops into aphorisms cautionary and otherwise, such as "domestic water should never be drawn from the heating system" or "in small cottage hospitals the administrative accommodation is usually provided in the same buildings as the wards"! In discussing cost, Mr. Wilson states that "a two-one bed hospital, including furnishing, will cost £300 per bed, and for a two-two bed hospital the cost will be about £180 per bed." This statement is entertaining, and reminds us of *Mr. Punch's* character who "whistled twice once." We must confess we can find little of permanent value in the additional construction chapter. In any future edition a chapter on hospital planning as affected by the great war, by Colonel Mackintosh, would have an added value as being the result of his present experience and observation. It might with advantage take the place of that just dealt with.

**A Manual for Nurses.** By Sydney Welham, M.R.C.S. Second Edition. (London: Mills and Boon. 1916. Pp. 230. Price 1s. 6d. net.)

The new edition of this manual follows pretty closely along the general lines of the first edition. It does not aspire to be a text-book of nursing, but simply to enable the nurse to deal more intelligently with particular cases of illness by providing her in handy form with a brief and clear synopsis of the principal features of the various diseases. In this aim it succeeds well. The general scheme is sound and well thought out; and though in a few minor points some people may not quite see eye to eye with Mr. Welham, on broad lines there can be little but general approbation of his manual.

**The Health of the Skin.** By George Pernet, M.D. (London: Methuen and Co. 1916. Pp. 106. Price 1s. net.)

The lay reader, for whom this book is clearly intended, will certainly, if he is at all intelligent, derive much quiet amusement from the pages of this unconventional text-book, as well as a good deal of instruction dexterously conveyed in an amusing form. Dr. Pernet is obviously a man of wide reading as well as an attentive and slightly cynical observer of human folly and frailty. In between his sly hits at poor humanity he manages to sandwich a great deal of sound advice on the care of the skin. Best of all, he has not attempted to provide the slightest encouragement for the self-treatment lunatic. He also avoids anything likely to drive nervous or morbidly sensitive individuals into panic. Altogether he has written a guide-book of many excellencies, which are rare enough in combination to merit a special note of appreciation.

**The Primary Lung Focus of Tuberculosis in Children.** By Anthon Ghon. Authorised Translation by D. Barty King, M.A., M.D., M.R.C.P. Illustrated. (London: J. and A. Churchill. 1916. Pp. 172. Price 10s. 6d.)

This monograph by the Professor of Pathological Anatomy at the University of Prague gives very voluminous details of an investigation into the relation of the primary focus of pulmonary tuberculosis in children to changes in the corresponding lymph-glands. As far back as 1876 Parrot enunciated a "law" which described the tracheo-bronchial lymphatic glands as a mirror of the lungs. Dr. Ghon's laborious researches tend to justify the assumption that in childhood the primary infection of the lungs represents the usual form of tuberculous infection. He also accepts the view that the aerogenous channel is the commonest path of infection. The main purpose, however, of this monograph is to prove that if any of the lymphatic glands in and around the root of the lung are found to be the seat of tubercular changes, then *careful* search will disclose a focus of disease in the lung, and in that portion of the lung which would be drained by the particular gland or set of glands in question. This research, to which it is impossible here to do justice, confirms the work of Kuss and of Albrecht, and will be of great interest to radiographers and the exponents of the retrograde lymphogenous origin of pulmonary tuberculosis in children. Incidentally, the specific value of the tuberculin reaction for diagnostic purposes receives support from Dr. Ghon. The very keen interest taken by Dr. Barty King in the subject of the origin of lung tuberculosis in children is well known to workers at the City Road Chest Hospital and at the Fleet Sanatorium. He has rendered a service to students of tuberculosis in making available in English this somewhat formidable treatise, which is, like most German monographs, rather verbose. The translator has kept, perhaps, unnecessarily close to the original text; some compression would have been a distinct advantage.

**Physiology for Nurses.** By W. B. Drummond, M.B., F.R.C.P. (Ed.). (London: Edward Arnold. 1916. Pp. 210. Price 2s. 6d. net.)

This elementary introduction to physiology is well arranged and judiciously written in all respects. We do not remember a better book for the instruction of nurses in that amount of physiology which it is desirable and necessary for them to know in order to grasp the meaning and *rationale* of many things dealt with in their work. The illustrations are adequate, even if some of them are a trifle crude in execution. Altogether, the book is one that can be strongly recommended to those for whom it is intended. The one drawback is the use of (in the opinion of the reviewer) a rather unlovely fount of type: but perhaps Mr. Arnold will not agree with this criticism, and such things are admittedly matters of opinion.

**Post-Mortem Methods.** By J. Martin Beattie, M.A., M.D. (Cambridge Public Health Series.) Illustrated. (Cambridge: University Press. 1915. Pp. 231. Price 10s. 6d.)

Inadequate and often useless records of *post-mortems* are the despair of the research worker or the statistician; on the other hand, an autopsy systematically performed and recorded in an orderly manner is a blessing to him who does it and to those who follow. However much it may be deplored in the abstract, the general practitioner is not likely to get any but infrequent opportunities for *post-mortem* examinations. It is therefore important that sections done in hospitals, especially the smaller ones, should be made the most of. Dr. J. M. Beattie's manual is one which might well find a place in hospital libraries, for it will prove of material help to those who are not specialists in pathology—particularly to house surgeons and to the staffs of country institutions. Like other members of the Cambridge Public Health series, it is clear and practical. The most advantageous methods of doing the actual cutting are described lucidly and accurately. Then follows the microscopical examination of the organs *seriatim*; next comes a chapter on necropsies for medico-legal purposes (chiefly poisonings), and then the author describes bacteriological methods as applied to special conditions. An appendix gives instructions for the preservation in their natural colours of museum specimens—Frost's solution being favoured for the final mounting. Notes on media and staining are inadequate compared with the rest of the book. It is interesting to find that this author is not at all keen on gloves as a protection; he advances cogent reasons for his attitude and his preference for running water. Still, he might have seen to it that the illustrations did not show an operator's hand with a ring retained.

**Maternity. Letters from Working Women.** (London: G. Bell and Sons. 1915. Pp. 212. Price 2s. 6d. net.)

The Women's Co-operative Guild circulated a year ago among a few hundred of its past and present officials (married women of the labouring classes) inquiries concerning their experiences of pregnancy and maternity; and from among 400 replies 160 letters have been selected for reproduction. In a preface which contains comments upon various social and economic aspects of maternity, the marriage laws, and infant mortality the writer asks, "What is the general impression that the reader gets of the life at such times of these more fortunate working-class mothers? It is on the whole an impression of perpetual overwork, illness, and suffering." Most certainly it is; and far be it from anyone interested in social problems to deny that much avoidable misery and suffering exist all over the kingdom amongst mothers and infants of the poorer classes. Poverty, ignorance, dirt, drink, malnutrition, and a score of evil influences which could be and must be mitigated or abolished are largely responsible for a state of affairs which shocks not only the members of the Women's Co-operative Guild but every thoughtful and instructed citizen, and gravely threatens the future of the State itself.

Admitting all that—and The Hospital has taken a humble part in advocating many of the measures already adopted for combating these evils—there is yet room for diversity of opinion as to the general line of future progress. It *is* true, undoubtedly, that the reader of this book will receive the painful impression claimed in the preface. But are the data on which those impressions are formed trustworthy? Do the letters from working women speak the truth, and nothing but the truth, or not? To the instructed reader there is scarcely one which is not tinctured with demonstrable inaccuracy. The most absurd statements, grotesquely impossible, are freely bandied in many of them; on internal evidence alone a fair proportion of them stand condemned as partly untrue, and therefore misleading. We say deliberately that the advocacy of a great cause by printing statements which contain so considerable an admixture of error, but are put before the general public on the ground that they are wholly true and trustworthy, is not the way to advance that cause or to enhance the reputation of the Women's Co-operative Guild.

Nevertheless, there is value even in the errors and garbled truths of the letters, as well as in the unexceptionable parts of them; for they do show what certain selected working women believe and think on these subjects; and such knowledge is highly important when remedies are propounded. If ignorance and prejudice are disclosed in them, as well as courage, affection, suffering, poverty, sickness, at least an indication is afforded as to factors in the problems of social betterment which might otherwise escape notice. In the propagandist part of the preface it is easy to gather some of the measures which the Guild would put in force to remedy glaring ills connected with material and infant welfare. We trust the Guild will not regard us as obstructionist or reactionary when we say that not all their proposals command our approval; and that they seem to us to want greater breadth of view and a better grasp of fundamental principles. With their objects we have the profoundest sympathy; with some of their methods we disagree, and with what we regard as an attempt to deceive public opinion we have no patience whatever.

